# Cohort Profile: Shanghai University of Sport (SUS) Longitudinal Cohort Study of Alumni Health Tracking

**DOI:** 10.2188/jea.JE20240494

**Published:** 2026-08-05

**Authors:** Huanyu Wang, Ruwen Wang, Qin Sun, Shanshan Guo, Kaiqing Lin, Qianqian Tian, Keneilwe Kenny Kaudimba, Mingjia Yang, Tiemin Liu, Ru Wang

**Affiliations:** 1 School of Exercise and Health, Shanghai University of Sport, Shanghai, China; 2 School of Life Sciences, Fudan University, Shanghai, China; 3 National Clinical Research Centre for Metabolic Diseases, The Second Xiangya Hospital of Central South University, Changsha, Hunan, China; 4 Metabolic Syndrome Research Centre, Key Laboratory of Diabetes Immunology, Ministry of Education, and Department of Metabolism and Endocrinology, The Second Xiangya Hospital of Central South University, Changsha, Hunan, China; 5 Xiangya School of Public Health, Central South University, Changsha, Hunan, China; 6 CSU-Sinocare Research Centre for Nutrition and Metabolic Health, Changsha, Hunan, China; 7 China Hospital Development Institute, Shanghai Jiao Tong University, Shanghai, China; 8 State Key Laboratory of Genetic Engineering, Department of Endocrinology and Metabolism, Institute of Metabolism and Integrative Biology, Human Phenome Institute, School of Life Sciences, Zhongshan Hospital, Fudan University, Shanghai, China; 9 School of Life Sciences, Inner Mongolia University, Hohhot, Inner Mongolia, China

**Keywords:** longitudinal cohort study, non-communicable diseases (NCDs), early adulthood, modifiable risk factors, multi-omics technologies

## Abstract

**Background:**

The Shanghai University of Sport (SUS) Longitudinal Cohort Study (SUS Cohort) was established to investigate the influence of lifestyle and modifiable behavioral factors in early adulthood on the development of non-communicable diseases (NCDs) later in life. The cohort aims to identify novel biomarkers and explore mechanisms underlying chronic diseases using a multi-omics approach.

**Methods:**

The SUS Cohort study is a prospective cohort study of undergraduate students between 2018 and 2019, with follow-ups conducted every 2 years during early adulthood and every 10 years thereafter. Comprehensive data collection includes body composition measurements, lifestyle surveys, physical fitness tests, and clinical laboratory tests. Multi-omics analysis, including genome-wide genotyping, gut microbiome, and serum metabolome, is integrated to provide insights into disease pathophysiology.

**Results:**

A total of 1,758 participants aged 18 to 22 (mean age 18.64; standard deviation, 3.15) years were conducted from baseline surveys, with a follow-up period between September 2021 and September 2022. The median age at baseline was 18 years, and female participants accounted for 49.5% of the cohort (*n* = 870). Of the initial participants, 1,055 individuals (60%) completed the follow-up.

**Conclusion:**

The SUS Cohort provides multidimensional data to study how early-life factors influence long-term health outcomes.

## PURPOSE

Early adulthood is a critical transitional period from late adolescence to adulthood that generally occurs between the ages of 18 and 25 years.^1^^,^^2^ During this stage, individuals experience a range of significant physical, behavioral, emotional, and social development.^3^^,^^4^ For example, a cross-sectional study comprising 1,095 Dutch college freshmen reported that 40% of the participants changed their eating behaviours and that 30.7% consumed more alcohol than they did previously.^5^ Furthermore, a longitudinal study of 250 first-year undergraduate students (88 men; mean age at initial assessment, 19.2; standard deviation [SD], 2.6 years) in the United Kingdom reported notable increases in body mass index, waist circumference, and fat mass after the first 3 months of university.^6^ Additionally, physical activity patterns can also be disrupted in early adulthood by the transition to college.^7^

The projected increase in non-communicable diseases (NCDs) is expected to lead to a significant increase in the number of deaths worldwide, from 60.1 million in 2022 to 90.7 million by 2050.^8^ The burden of NCDs attributable to both premature death and years spent in ill health is expected to increase from 33.8% to 41.1%, indicating a shift in the global disease burden toward chronic morbidity and long-term disability.^8^ As a crucial turning point for several chronic disease-related risk factors, early adulthood may be an important window for intervention to prevent chronic diseases and premature death. Multiple studies, including studies of United States, European, and China Kadoorie Biobank cohorts, have shown that overweight and obesity in early adulthood may increase the risk of developing major chronic diseases in middle-aged and elderly individuals, including all-cause mortality, cardiovascular disease, and type 2 diabetes.^9^^–^^12^ In a prospective study of males (mean age, 22 years), a 36 mg/dL (0.9 mmol/L) increase in baseline serum cholesterol was associated with a 72% higher risk of cardiovascular disease and a 102% increase in mortality.^13^ However, there is limited evidence on the long-term effects of lifestyle factors during early adulthood on other chronic diseases and the mechanisms underlying these effects.

## MAIN FEATURES

The Shanghai University of Sport Longitudinal Cohort Study of Alumni Health (SUS Cohort) was established to investigate how lifestyle, behavioral, and physical health factors during early adulthood affect the long-term risk of NCDs. The SUS Cohort is distinctive in its comprehensive, multidimensional approach to data collection and long-term follow-up design. At the baseline and follow-up visits, extensive data were collected from the cohort through standardized procedures, including electronic questionnaires, anthropometric and body composition measurements, physical fitness testing, and biospecimen sampling for laboratory analyses. Additionally, accelerometer-based activity tracking was incorporated to provide objective measures of physical activity and sedentary behavior.

**Figure 1.**
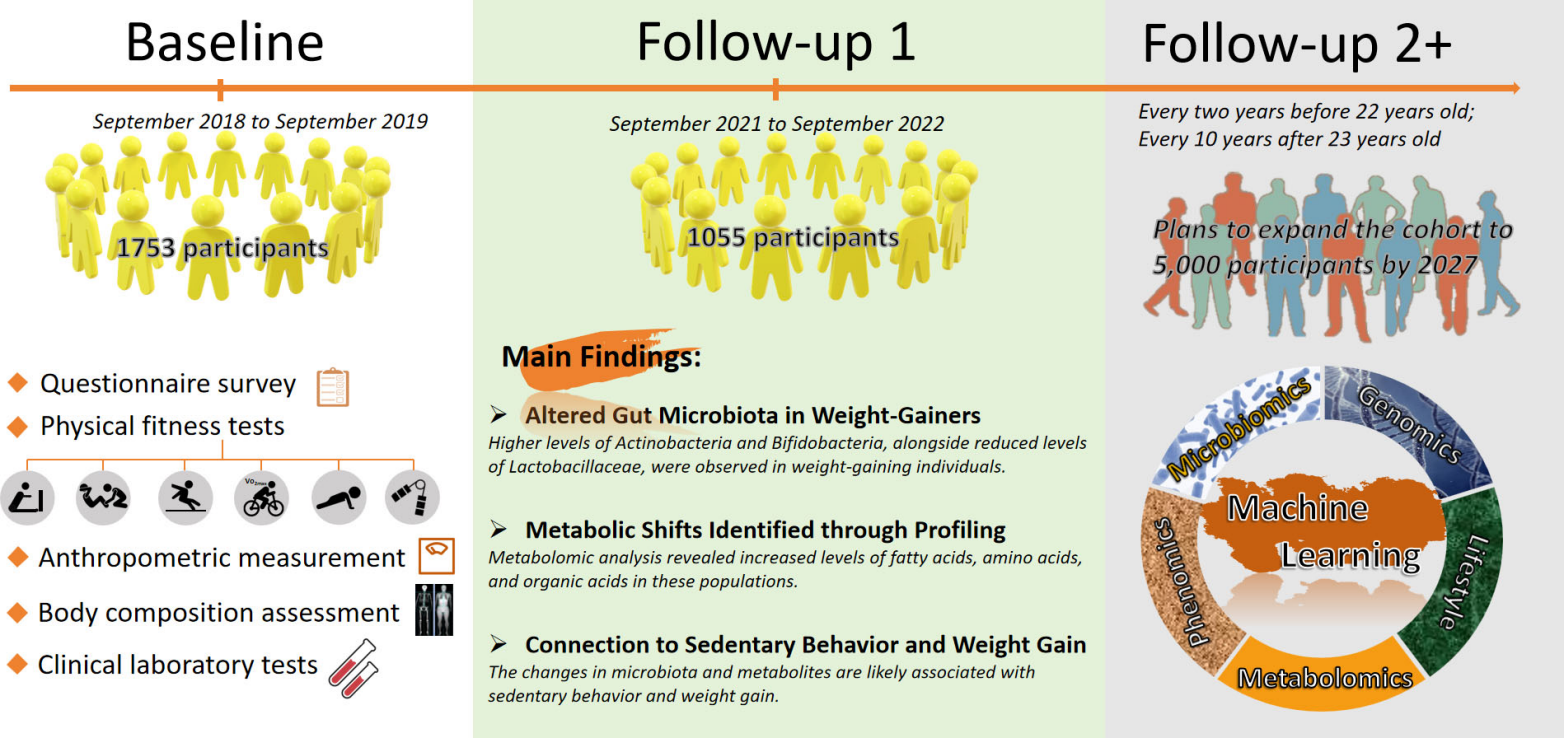
Study design of the SUS cohort

Importantly, the SUS Cohort integrates multi-omics platforms, including genome-wide genotyping, gut microbiome sequencing, and targeted metabolomics, to identify novel biomarkers and mechanisms underlying disease development. This life-course approach enables the exploration of both short-term changes and long-term health trajectories. Figure 1 provides a visual overview of the structure and timeline of the SUS Cohort study.

The core objectives of this cohort are threefold:

(1) to identify modifiable lifestyle and behavioral factors in early adulthood that are associated with NCDs later in life;
(2) to discover and validate omics-based biomarkers and underlying mechanisms for NCDs;
(3) to construct predictive models for long-term health outcomes based on both behavioral and molecular indicators.

### Participants

All participants in the SUS Cohort were recruited from Shanghai University of Sports. A total of 1,753 undergraduate students between the ages of 18 and 22 years were enrolled from September 2018 to September 2019; these students were from 34 provinces in China. The study participants were included if they (i) were first-year undergraduate students at SUS who enrolled in 2018 or 2019; (ii) were ≥18 years old; and (iii) signed an informed consent from to participate in the study. Participants were excluded if they: (i) had taken antibiotic medications within one week of biosample collection; (ii) had been diagnosed with type 2 diabetes, hypertension, hypothyroidism, hyperthyroidism, or other endocrine hormone abnormality-related diseases; or (iii) had consumed functional foods, protein powders, or medications within the last 6 months.

### Outcomes and follow-up

Baseline data collection for the SUS took place from September 2018 to September 2019. Participants were scheduled for biennial (every 2 years) follow-up during early adulthood (ages 18–22 years) and every 10 years thereafter (from the age of 23 years onward), with planned follow-up extending at least through middle adulthood (up to the age of 60 years). The detailed selection and exclusion process are illustrated in eFigure 1. A total of 1,055 individuals were followed up from September 2021 to September 2022, with a response rate of 60%. This longitudinal follow-up scheme enables comprehensive analysis of lifestyle, physical fitness, and metabolic risk transitions across the lifespan.

The primary outcomes of the SUS Cohort include the incidence of major NCDs, such as type 2 diabetes, hypertension, dyslipidemia, cardiovascular diseases (eg, coronary heart disease and stroke), and cancer, as well as all-cause mortality. During follow-up, participants are first contacted directly via phone or social media platforms such as WeChat, QQ, or their parents’ phone numbers. In addition, outcome information on NCDs is obtained through data linkage with national surveillance systems, including the chronic disease registry and death registry maintained by the Chinese Center for Disease Control and Prevention. This combination enhances both the completeness and reliability of the outcome data.

In the second follow-up phase (planned after 2024), machine-learning approaches will be applied to integrate longitudinal lifestyle data, physical measurements, and multi-omics profiles to predict the NCD risk trajectories. Supervised models (eg, random forest, support vector machine) and unsupervised clustering methods will be used to identify high-risk subgroups and derive data-driven health prediction models.

### Measurement

A face-to-face questionnaire survey, physical fitness tests, anthropometric measurement, body composition assessment and clinical laboratory tests were conducted in this study. Meanwhile, the same measurements were re-examined at follow-up examination. The entire visit procedure, including the electronic questionnaire, medical examination, and sample collection, typically took 120 minutes per participant to complete. All study investigators had a medical background and were trained and evaluated after training to be qualified for working on the specific study tasks.

#### Questionnaire

The face-to-face questionnaire interview collected baseline information including demographic and socioeconomic characteristics, environmental exposures, lifestyle factors, medical history and level of physical activity using an online application called “SoJump” (detailed in Table 1). The questionnaire referred to the UK Biobank as a reference, and validated instruments for food frequency (Food Frequency Questionnaire) and quality of life were used. The design of the questionnaire was informed by the structure of the UK Biobank baseline questionnaire.^14^ Dietary intake was assessed using a food frequency questionnaire (FFQ) specifically developed for this study, based on commonly used formats in Chinese nutritional research.^15^ Quality of life was evaluated using the validated 12-item short-form health survey.^16^

**Table 1.** Information collected in the questionnaire survey

Questionnaire survey	Number of variables	Description
Demographic and socio-economic information	10	Sex, date of birth, name, age, height, Parents’ education, parents’ occupation
Environmental exposure	69	Objective environment, building environment
Emotional, Psychological, Anxious	57	Life satisfaction, mood status, Emotional processing abilities and strategies, quality of life (SF-12)
Lifestyle	129	Diet (including Food Frequency Questionnaire), physical activities (IPAQ-12), sleeping habits (PSQI)
Medical history	10	Personal and family disease history, health status

IPAQ-12, 12-item International Physical Activity Questionnaire; PSQI, Pittsburgh Sleep Quality Index; SF-12, 12-item Short Form Health Survey.

#### Anthropometric measurement and body composition assessment

Height and weight were measured using the TANITA anthropometer (model 46443a; Tanita Corp, Tokyo, Japan). Height was measured to the nearest 0.1 cm (1 mm) and weight to the nearest 0.1 kg (100 g). Each measurement repeated twice by the same staff member; the mean values were then calculated for data analysis. Body mass index (BMI) was calculated as weight (kg) divided by height (m^2^). Systolic and diastolic blood pressure measurements were taken twice using a sphygmomanometer with a weighted blood pressure cuff, with a 3-minute interval between measurements. In addition, mean arterial pressure was calculated using the formula: mean arterial pressure = (systolic + 2 * diastolic)/3. Components of body composition—including bone mass composition, body fat, and lean/muscle mass—were measured using dual-energy X-ray absorptiometry (DXA). Once the test is complete, the system’s built-in software calculates body fat, muscle mass, and bone density for the whole body as well as for each part of the body. Body fat percentage (%) = Total body fat/(Total body muscle + Total body fat) * 100%; Android fat percentage (%) = Android fat/(Android muscle + Android fat) * 100%; Gynoid fat percentage (%) = Gynoid fat/(Gynoid muscle + Gynoid fat) * 100%.

#### Physical fitness

Physical fitness was evaluated using a Physical-Fitness Test System (Takei Kiki Kogyo, Tokyo, Japan). Participants were instructed to rest for 5 minutes before each test.

Upper body muscular strength (UBMS) was assessed using a handgrip test, performed with a hand dynamometer featuring an adjustable grip. The test was administered twice for each hand, recording the highest score in kilograms. The average of these maximum scores was calculated for analysis.

Lower body explosive strength was assessed using the standing long jump test. The test was conducted twice, recording the maximum distance for analysis.

Whole body reaction time was used to assess the participant’s sensitivity and speed in response to visual stimuli. It specifically measured the time taken to lift a leg from a starting platform. Reaction times were recorded across five trials with precision up to 0.001 seconds for analysis.

Hamstring flexibility was assessed using a sit-and-reach test. Participants sat with knees fully extended and feet against a vertical support, reaching forward with both hands. Two attempts were recorded, and the highest scores were used for analysis, measured to the nearest 0.1 cm.

The balance of the subjects was assessed using standing on one leg for as long as possible with eyes closed. Each participant made two attempts, and the longer duration was recorded with an accuracy of 1 second.

Cardiorespiratory fitness was assessed using a power bike test to achieve reach maximal oxygen consumption (VO_2_max) (MATRIX Active Cycle U1X; Johnson Fitness and Wellness, Osaka, Japan). Participants were instructed to start pedaling the power bike rhythmically, keeping the speed at around 50 rpm. The machine increases the load at each 3-minute period, until 13-minute period the test is completed. The test was finished when the machine’s in-built software automatically calculates the maximum oxygen uptake value to the nearest 0.1 mL/kg/min.

Abdominal endurance was assessed using 1-min sit-ups. Subjects performed a full sit-up to the upright position with their elbows touching their thighs and then returned to the supine position where their shoulders (scapula) touched the mat surface. The number of repetitions in 60 s was counted.

Physical activity (PA) and sedentary time was assessed by wearing an innovative triaxial accelerometer (ActiGraph^®^, Model GT3X; ActiGraph, Pensacola, CA, USA) for 7 consecutive days after the clinical visit. Participants underwent 7 consecutive days of wearing the accelerometer on the left side of the waist (except during swimming, bathing and sleeping hours). Participants with a valid wearing time of 480 minutes or more on the day of wearing were considered eligible data, and those less wearing time were excluded.^17^ The measure of physical activity in young adults was divided into the four categories of (1) sedentary time, (2) light PA, (3) moderate PA, and (4) vigorous PA, and is based on the cutoff points of 0–99, 100–2,000, 2,001–5,724, and >5,724 counts/min, respectively.^18^ The study categorized physical activity as moderate-to-vigorous (MVPA, >2,000 counts/min) and sedentary time as less than 100 counts/min.

### Biological sample collection and storage

Fasting blood, urine and fecal samples were collected. Fasting blood samples were obtained by venipuncture after an overnight fast (average fasting time, 10.75 hours, standard deviation [SD], 5.3 hours); 15 mL of blood was collected from each individual and divided into 3 different blood collection tubes: 5 mL of serum, 5 mL of sodium fluoride anticoagulant tube and 5 mL of ethylenediaminetetraacetate EDTA anticoagulant tube. Urine samples were collected in sterile containers during the morning visit after an overnight fast. Fecal samples were self-collected by the participants using provided sterile collection kits. The fecal samples were evenly divided into three 5 mL sterile cryovials. All collected samples were initially stored at −20°C and then transported to the laboratory, where they were stored at −80°C until analysis (detailed in Table 2).

**Table 2.** Information collected on blood biochemistry

Laboratory Testing	Method/Analyzer	No. of Variables	Description
Haematology	Sysmex XE-2100 Analyzer	12	HGB, HCT, MCV, MCH, MCHC, RDW-CV, RDW-SD, PLT, MPV, PDW, P-LCR, PCT
Glucose	Roche Cobas C8000 Analyzer	2	Fasting blood glucose, HbA1c
Blood lipids	Chemical method, Immune turbidimetry	6	TC, TG, HDL-C, LDL-C, APO-AI, APO-B
Trace elements	Atomic absorption spectrometry	7	Zn (µmol/L), Mg (mmol/L), Fe (mmol/L), Pb (µg/L), Cd (µg/L), Ca (mmol/L), Cu (µmol/L), Vitamin D
Renal function	Chemical method	3	Urea, Creatinine (Cr), Uric acid (UA)
Gut microbiome	DNA Nanoball Sequencing	1,747	41 Classes, 89 Orders, 225 Families, 571 Genera, 1,302 Species, 1,398 Strains
Targeted Metabolomics	UPLC-MS/MS	209	Amino acids, Benzenoids, Benzoic acids, Nucleotides, Carbohydrates, Carnitines, Fatty acids, Indoles, Nucleotides, Organic acids, Peptides, Phenols, Phenylpropanoid acids, Phenylpropanoids, Pyridines, SCFA
Genome	DNA Nanoball Sequencing	Almost 3,000,000 SNPs	SNPs, Indels, SVs, CNVs

APO-AI, apolipoprotein AI; APO-B, apolipoprotein B; Ca, calcium; Cd, cadmium; CNVs, copy number variations; Cr, creatinine; Cu, copper; DNA, deoxyribonucleic acid; Fe, iron; HbA1c, glycated hemoglobin; HCT, hematocrit; HDL-C, high-density lipoprotein cholesterol; HGB, hemoglobin; LDL-C, low-density lipoprotein cholesterol; MCH, mean corpuscular hemoglobin; MCHC, mean corpuscular hemoglobin concentration; MCV, mean corpuscular volume; Mg, magnesium; MPV, mean platelet volume; PCT, plateletcrit; PDW, platelet distribution width; P-LCR, platelet-large cell ratio; PLT, platelet count; RDW-CV, red cell distribution width coefficient of variation; RDW-SD, red cell distribution width standard deviation; SCFA, short-chain fatty acids; SNPs, single nucleotide polymorphisms; SVs, structural variations; TC, total cholesterol; TG, triglycerides; UA, uric acid; UPLC-MS/MS, ultra-performance liquid chromatography tandem mass spectrometry; Zn, zinc.

## BASELINE CHARACTERISTICS

The sociodemographic characteristics of the study participants at baseline are presented in Table 3. A total of 1,753 participants were included in the analysis, of whom 871 (49.7%) were women. The mean age was 18.64 (SD, 3.15) years, with similar age distributions for men (mean 18.73; SD, 1.06 years) and women (mean 18.55; SD, 4.86 years; *P* = 0.289, *t*-test). Further stratification by BMI indicated that underweight was more prevalent among men, whereas overweight and obesity were more common among women (*P* < 0.001, chi-square test). Anthropometric and blood pressure measurements revealed that, compared with females, males presented greater systolic and diastolic blood pressures, as well as greater waist circumferences (all *P* < 0.001). In terms of physical fitness, males demonstrated significantly greater average handgrip strength, greater maximal oxygen uptake, and better performance in sit-ups, push-ups, and standing long jumps (all *P* < 0.001). Conversely, females exhibited superior flexibility, as evidenced by higher sit-and-reach scores (*P* < 0.001). Body composition assessments (via DXA) demonstrated that males had significantly greater android and gynoid fat masses than females did (*P* < 0.001 for both). Blood biochemical indices also exhibited marked sex differences: men presented higher levels of triglycerides and apolipoprotein B, whereas women presented higher high-density lipoprotein cholesterol and apolipoprotein A1 concentrations (all *P* < 0.001). No significant difference in low-density lipoprotein cholesterol levels was observed. The glucose levels were similar between the groups, but the hemoglobin, alanine aminotransferase, creatinine, uric acid, and red blood cell counts were significantly greater in men (all *P* < 0.001), whereas plateletcrit was greater in women (*P* < 0.001).

**Table 3.** Baseline characteristics of the study cohort

Characteristics	Male (*n* = 882)	Female (*n* = 871)	Total (*n* = 1,753)	*P*-value
Age, years	18.73 ± 1.06	18.55 ± 4.86	18.64 ± 3.49	0.289^†^
BMI, kg/m^2^	21.71 ± 3.18	21.23 ± 3.45	21.47 ± 3.32	0.002^†^
BMI categories, *n* (%)				<0.001^‡^
Underweight	290 (32.88)	81 (9.30)	371 (21.16)	
Normal weight	488 (55.33)	496 (56.95)	984 (56.13)	
Overweight	81 (9.18)	163 (18.71)	244 (13.92)	
Obesity	23 (2.61)	131 (15.04)	154 (8.79)	
SBP, mm Hg,	124.13 ± 14.02	110.43 ± 12.82	117.4 ± 15.09	<0.001^†^
DBP, mm Hg	72.93 ± 11.71	68.46 ± 10.17	70.74 ± 11.2	<0.001^†^
HR, bpm	81.00 ± 14.02	85.56 ± 13.64	83.24 ± 14.01	<0.001^†^
WC, cm	77.90 ± 9.57	72.00 ± 9.58	75.04 ± 10.02	<0.001^†^
Physical fitness				
Average handgrip strength, kg	41.43 ± 6.28	25.94 ± 4.6	33.82 ± 9.51	<0.001^†^
Sit and reach, cm	14.60 ± 7.71	17.28 ± 7.27	15.92 ± 7.61	<0.001^†^
Maximum oxygen intake, mL kg^−1^ min^−1^	42.75 ± 8.44	33.45 ± 7.34	38.30 ± 9.19	<0.001^†^
Sit-ups, min^−1^	46.18 ± 12.35	37.94 ± 12.46	42.14 ± 13.07	<0.001^†^
Push-ups, min^−1^	48.45 ± 18.39	22.41 ± 17.66	35.67 ± 22.24	<0.001^†^
Standing long jump, cm	241.24 ± 26.56	175.37 ± 24.9	208.8 ± 41.81	<0.001^†^
Android FM, kg	67.05 ± 15.32	55.69 ± 10.77	61.54 ± 14.51	<0.001^†^
Gynoid FM, kg	318.34 ± 70.30	223.04 ± 44.05	271.97 ± 76.08	<0.001^†^
Lipid associated				
TG, mmol L^−1^	0.92 ± 0.53	0.81 ± 0.35	0.87 ± 0.45	<0.001^†^
HDL-C, mmol L^−1^	1.21 ± 0.21	1.38 ± 0.23	1.29 ± 0.24	<0.001^†^
LDL-C, mmol L^−1^	2.23 ± 0.55	2.25 ± 0.52	2.24 ± 0.53	0.572^†^
APOA, g L^−1^	1.41 ± 0.24	1.44 ± 0.24	1.42 ± 0.24	0.007^†^
APOB, g L^−1^	0.82 ± 0.17	0.78 ± 0.16	0.8 ± 0.16	<0.001^†^
Glucose associated				
Glucose, mmol L^−1^	4.44 ± 0.41	4.47 ± 0.4	4.46 ± 0.41	0.210^†^
Hb, g L^−1^	153.57 ± 12.64	132.95 ± 10.68	143.31 ± 15.6	<0.001^†^
Blood chemistry				
ALT, umol L^−1^	21.71 ± 17.83	14.27 ± 11.76	18.01 ± 15.57	<0.001^†^
Creatinine, umol L^−1^	88.47 ± 14.5	67.87 ± 13.86	78.22 ± 17.53	<0.001^†^
Uric acid, umol L^−1^	418.91 ± 91.04	311.98 ± 69.16	365.72 ± 96.96	<0.001^†^
Plateletcrit, %	246.82 ± 51.46	264.58 ± 49.76	255.66 ± 51.38	<0.001^†^
Red blood cell count, 10^12^ L^−1^	5.15 ± 0.43	4.54 ± 0.34	4.84 ± 0.49	<0.001^†^

APOA, apolipoprotein A-I; APOB, apolipoprotein B; ALT, alanine aminotransferase; DBP, diastolic blood pressure; FM, fat mass; Hb, hemoglobin; HDL-C, high-density lipoprotein; HR, heart rate; LDL-C, low-density lipoprotein; SBP, systolic blood pressure; TG, triglyceride; WC, waist circumference.

Values are presented as mean ± standard deviation (SD) for continuous variables or as number (percentage) for categorical variables. *P*-values were calculated using the independent *t*-test (†) for continuous variables and the chi-square test (‡) for categorical variables. For BMI, both the continuous measure (kg/m^2^) and categorical groups (underweight, normal, overweight, obese) are presented for clarity.

## MAIN OUTCOMES

In subsequent analyses among participants, normal-weight obesity (NWO) was linked to poorer fitness and increased cardiometabolic risk, with a 28% higher prevalence of NWO per one-unit increase in the metabolic syndrome score.^19^ The finding further demonstrated that greater physical fitness was associated with healthier gut microbiota and a lower risk of diabetes, although the abundance of *Akkermansia muciniphila* was paradoxically lower in fitter individuals.^20^ Over 2 years, individuals who gained weight presented increased body fat, decreased levels of glycemic markers, unfavorable gut microbiota shifts, and metabolomic changes dominated by fatty acids, amino acids, and organic acids.^21^

### Strengths and limitations

This prospective longitudinal study aims to explore the effects of both short-term and long-term lifestyle changes on health outcomes. Researchers have collected variables as well as biospecimens for longitudinal analysis. A wide range of opportunities are provided to link progressive changes in disease biomarkers and the development of diseases to variations in individuals’ phenotypes in terms of physical activity and lifestyle factors. In addition, the microbiomics, metabolomic, and genotype data could be used to investigate their direct or indirect effects on the development of NCDs.

Despite its strengths, the SUS Cohort has several limitations. First, the current sample size of approximately 1,500 participants may limit the statistical power of certain analyses, particularly those requiring subgroup comparisons. However, plans to increase the cohort to 5,000 participants by 2027 exist, which will help to address this issue. Another challenge is participant attrition during long-term follow-up, which may affect data completeness and study validity. Strategies such as regular participant engagement and retention incentives will be implemented to minimize loss to follow-up. Additionally, while the SUS Cohort consists of university students and thus may not be representative of the general population, it is designed to be internally representative of the Shanghai University of Sport undergraduate population. The high participation rate, inclusion of students from all disciplines, and wide geographical distribution of students enhance its representativeness within this academic setting. Efforts will be made to increase the cohort in the future to include a more diverse participant base.

## SUPPLEMENTARY MATERIAL

eFigure 1. Participant flow diagram of SUS cohort (2018–2022)
