# Supplementary material for: Cohort Profile: Shanghai University of Sport (SUS) Longitudinal Cohort Study of Alumni Health Tracking
Source: J Epidemiol. 2026 Aug 5;36(8):265–70. doi: 10.2188/jea.JE20240494 (PMC13349788; doi:10.2188/jea.JE20240494)
Supplement: Supplementary file 1 [file je-36-265-s001.pdf]

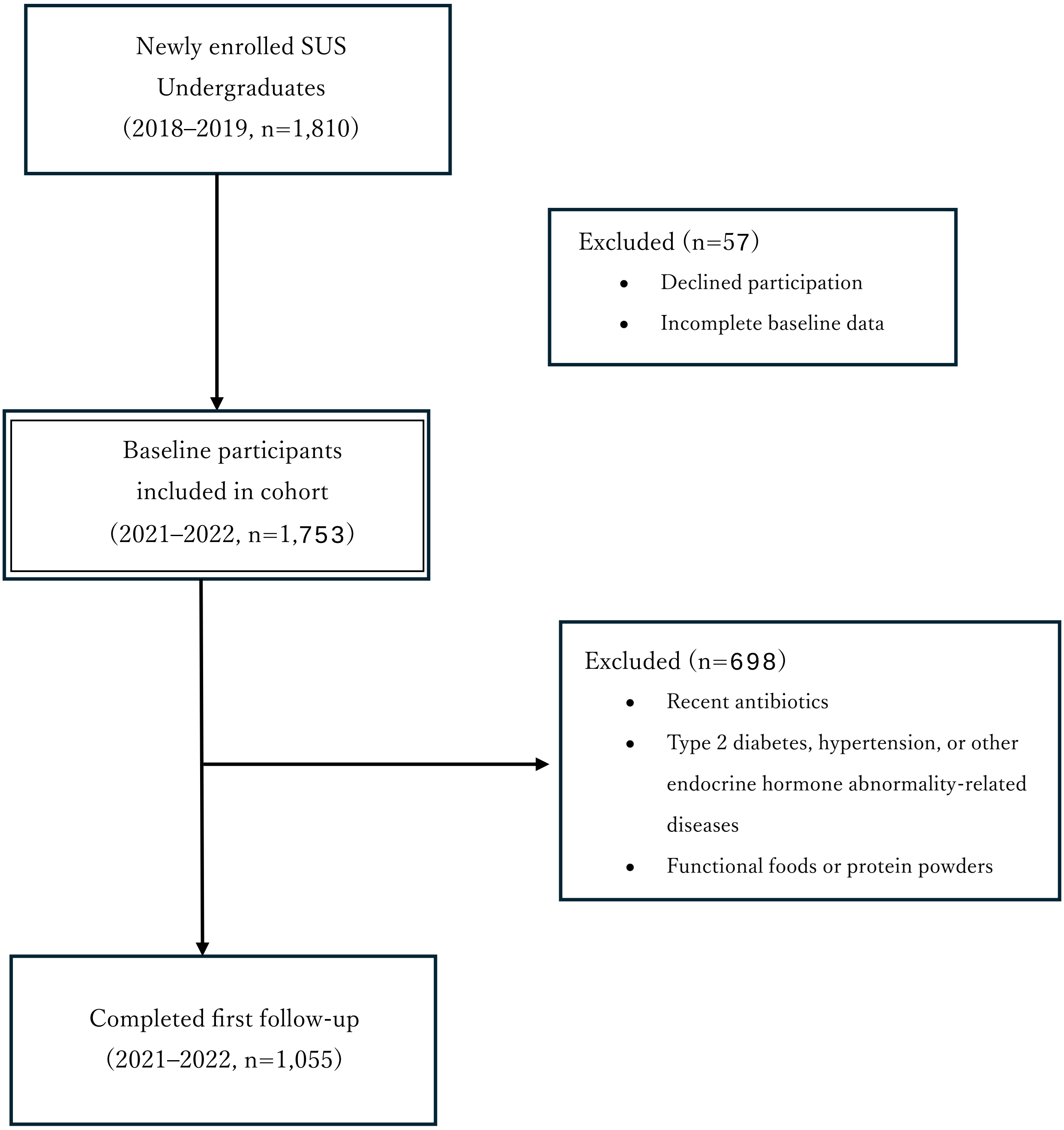

eFigure 1. Participant flow diagram of SUS cohort (2018–2022)
